# Do animal husbandry operations contaminate groundwater sources with antimicrobial resistance: systematic review

**DOI:** 10.1007/s11356-024-31899-w

**Published:** 2024-02-06

**Authors:** Cameron Meyer, Skyler Price, Ayse Ercumen

**Affiliations:** Department of Forestry and Environmental Resources, North Carolina State University, 2800 Faucette Dr, Raleigh, NC 27607 USA

**Keywords:** Food production animals, Animal feeding operations, Antibiotic resistance, Water quality, Private wells, Animal waste management

## Abstract

Antimicrobial resistance (AMR) is a critical global health concern. Animal husbandry operations are AMR hotspots due to heavy antibiotic use and dissemination of animal waste into the environment. In this systematic review, we examined the impact of swine, poultry, and cattle operations on AMR in groundwater. We searched PubMed, Web of Science, CAB Direct, and the North Carolina State University Agricultural and Environmental Science databases in June 2022. The search returned 2487 studies. Of the 23 eligible studies, 17 were conducted in high-income countries (primarily the USA, also Canada, Saudi Arabia, Cyprus), and 6 were conducted in a single upper-middle-income country (China). Studies investigated facilities for swine (13), poultry (4), cattle (3), and multiple types of animals (3). The sampling distance ranged from onsite to > 20 km from facilities; the majority of studies (19) sampled onsite. Most studies collected samples from monitoring wells; only 5 studies investigated private drinking water wells. AMR in groundwater was associated with animal husbandry operations in 74% (17/23) of all studies, 65% (11/17) of studies in high-income countries, and 100% (6/6) of studies in China. Contamination was mostly found in onsite wells, especially downgradient of waste lagoons, but also in offsite private wells up to 2–3 km away. Few studies reported weather data, but AMR contamination appeared to increase with rainy conditions. Future studies should sample private wells at varying distances from animal husbandry operations under different weather conditions and include low- and middle-income countries where food animal production is intensifying.

**Supplementary Information:**

The online version contains supplementary material available at 10.1007/s11356-024-31899-w.

## Introduction

Antimicrobial resistance (AMR) reduces the effectiveness of antibiotics, resulting in increased mortality, prolonged duration of infections, and increased healthcare costs (O’Neill 2016). Resistance can occur when humans/animals are frequently exposed to antibiotics, selecting resistant bacteria in the gut (Andersson and Hughes 2010). AMR can further emerge and spread when antibiotic residues, antimicrobial-resistant bacteria (ARB), and antimicrobial resistance genes (ARGs) from human/animal fecal waste are released into the environment. Under selection pressure from antibiotic residues and other biocides present in environmental compartments, fecal and environmental microorganisms can exchange ARGs via horizontal gene transfer (Huijbers et al. 2015).

Animal husbandry operations are a hotspot for AMR, both because of the heavy use of antibiotics in food production animals and the waste management practices that widely disseminate animal waste from these facilities into the surrounding environment. Animal husbandry operations encompass the breeding and production of animals for the purpose of harvesting animal products. Confined animal feeding operations (CAFOs) are larger operations that are classified by the number and type of animals and their waste management practices (Hribar 2010). Antibiotics are commonly administered to prevent disease or promote growth in the animals raised in animal husbandry operations, and CAFOs require more intensive antibiotic use per animal than smaller operations due to animal proximity and increased risk of infection transmission (Landers et al. 2012). Globally, 73% of all antimicrobials are consumed by food animals (Van Boeckel et al. 2019). As a result, antibiotic residues, ARB, and ARGs are frequently detected in animal waste from CAFOs (Burkholder et al. 2007).

A single CAFO can produce between 2800 and 1.6M t of manure per year (Hribar 2010). This waste is typically stored onsite and applied untreated to nearby farmlands (Burkholder et al. 2007). In the USA, swine CAFOs store their waste in earthen pits, referred to as waste lagoons, to allow it to degrade. The storage time may be sufficient to break down some antibiotics, while residues may remain for others, and ARB/ARGs may persist (Chee-Sanford et al. 2009; Hribar 2010). The contents of waste lagoons are then sprayed on nearby farmlands, and the sludge within the lagoons (the densest materials that deposit at the bottom) is dredged periodically and applied to croplands as fertilizer (Heuer et al. 2011). Poultry waste is collected in open heaps and also applied to land as fertilizer, and ARB/ARGs persist in the litter over typical storage durations (Graham et al. 2009b). These practices can result in the concentrated delivery of ARB and ARGs into environmental compartments. For example, gene copies of ARGs in soil have been shown to increase shortly after the application of manure to soil (Huijbers et al. 2015). Contaminants from CAFOs can reach nearby ambient waters and groundwater aquifers. Mechanisms for transport include runoff and infiltration from fields where manure has been applied, leaking or overflowing lagoons, vectors (e.g., flies), and bioaerosols generated during spraying (Graham et al. 2009a; Heuer et al. 2011; EPA 2013). These processes can occur chronically as well as episodically during extreme weather (Mallin et al. 2015; Harris et al. 2021). Private groundwater wells are particularly vulnerable because water quality from private wells is not regulated by the Safe Drinking Water Act (EPA 2015), and most well water is consumed untested and untreated (Gibson and Pieper 2017).

The demand for animal protein is expected to continue to rise, especially in low- and middle-income countries. With this increased demand comes a greater reliance on antimicrobials in larger-scale operations as animal husbandry operations become more concentrated for increased efficiency; consumption of antimicrobials by food animals is expected to increase 67% globally between 2010 and 2030 from 63,000 to 106,000 t in the absence of regulatory controls (Van Boeckel et al. 2015). Rates of AMR are also increasing globally, especially in low- and middle-income countries, both in humans and in food production animals (Van Boeckel et al. 2019; Murray et al. 2022). It is important to assess the impact of animal husbandry operations on the risk of exposure to AMR from groundwater resources. We conducted a systematic review to investigate associations between animal husbandry operations and the occurrence of ARB and ARGs in groundwater.

## Material and methods

### Literature search

We developed a list of search terms that covered synonyms for (i) animal husbandry, (ii) antimicrobial resistance, and (iii) groundwater. We generated one finalized search string by combining search terms with an “OR” operator within each of these categories and with an “AND” operator across the three categories (Text S1). We searched PubMed, Web of Science, CAB Direct, and Agricultural and Environmental Science databases for peer-reviewed literature and Science.gov for gray literature. The search was conducted in June 2022. We exported references identified in the search to the Mendeley reference manager where duplicates were removed. Using Covidence systematic review software, one researcher (CM) screened the titles and abstracts of each study and reviewed the full texts of shortlisted studies. For any eligible studies and review articles identified during the full-text review, we screened the bibliographies for additional relevant references.

### Criteria for inclusion and exclusion

We aimed to include studies that sampled groundwater in areas with active animal husbandry operations that raised or harbored swine, poultry, or cattle and reported a measure of antimicrobial resistance within groundwater samples. We only included studies that focused on areas with ongoing animal husbandry operations and excluded studies that solely focused on fields where manure was applied; this was intended to capture impacts from the full range of waste management processes (e.g., potential leakage from lagoons) associated with animal husbandry operations. We excluded experimental studies (e.g., studies where manure was applied to soil under controlled conditions). We only included studies in English.

### Data extraction and synthesis

Two researchers (CM, AE) independently extracted data from eligible studies and resolved any discrepancies by discussion. Using Microsoft Excel, we extracted data for the following characteristics: study year and location, type(s) and number of animals, groundwater sampling information (distance from animal husbandry operations, type and depth of wells, sampling frequency), methods used to assess antimicrobial resistance, prevalence and abundance of ARB/ARGs in samples, and other relevant environmental information (e.g., soil type, groundwater table, climate/weather conditions, and ARB/ARG detected in environmental samples other than groundwater). We also extracted information on any control sites (i.e., locations unimpacted by animal husbandry operations) sampled in the studies. Because ARB/ARGs in groundwater can stem from many sources, sampling at a control site can help isolate the impact of animal husbandry operations. We qualitatively synthesized data to assess the impact of animal husbandry operations on antimicrobial resistance in groundwater separately by animal type (swine, poultry, cattle, mixed) and by the income category of the country of study, as defined by the World Bank for the year that each study was published (Hamadeh et al. 2022). We further summarized findings by well proximity to animal husbandry operations and well type (e.g., monitoring well, domestic well). Our Preferred Reporting Items for Systematic Reviews and Meta-Analyses (PRISMA) checklist can be found in the Supplementary Information (Table S1).

## Results

### Literature search and screening

**Fig. 1:**
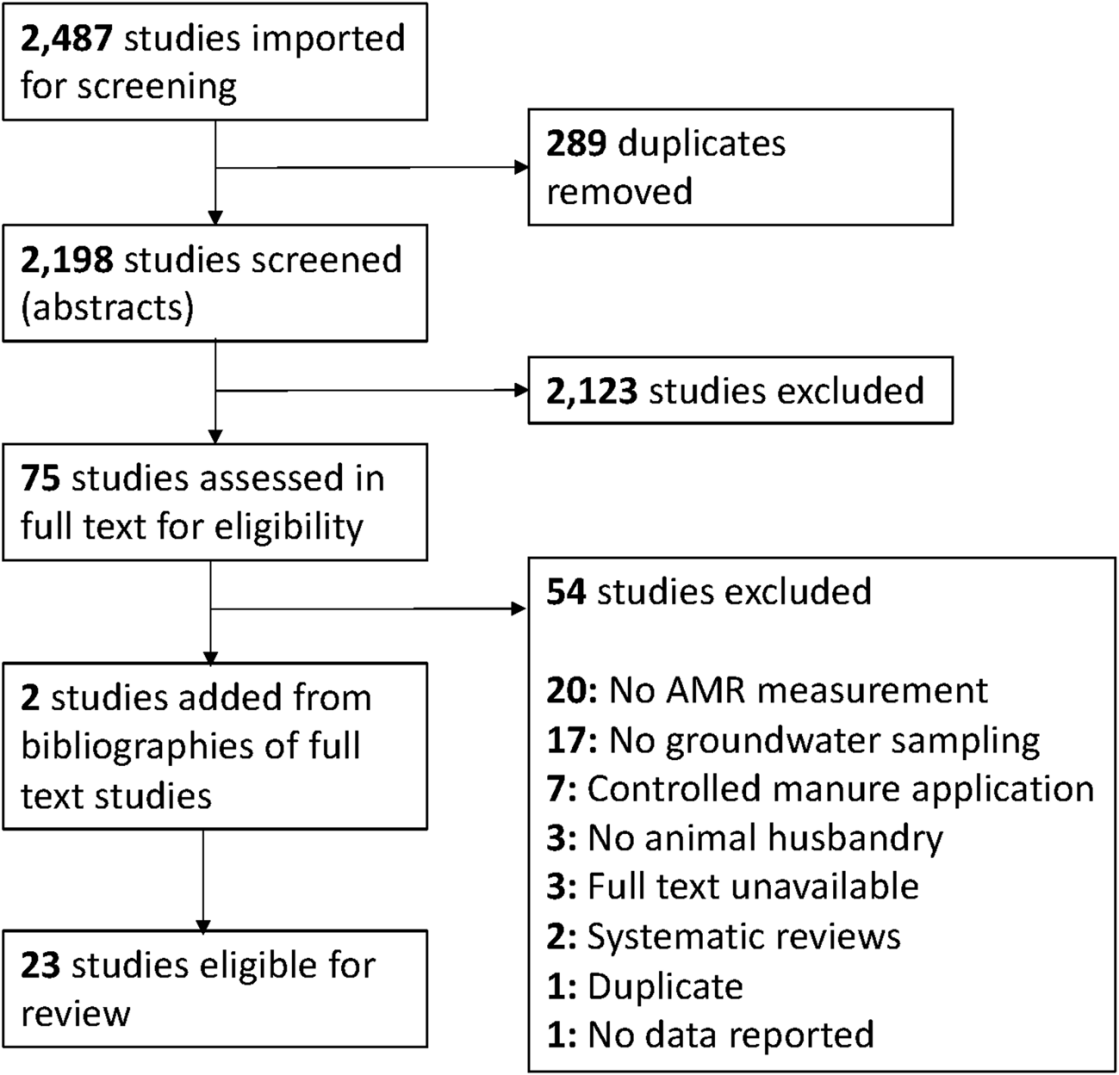
PRISMA flowchart for systematic review search and screening process

Our search returned a total of 2487 results, with 2198 unique studies after duplicates were removed (Fig. 1). We reviewed the full texts of 77 studies and identified 23 eligible studies based on our inclusion and exclusion criteria.

### Study characteristics

The 23 studies included in our review were conducted between 2001 and 2022. Seventeen studies were conducted in high-income countries (primarily the USA, also Canada, Cyprus, and Saudi Arabia), and 6 were conducted in China, which was the only upper-middle-income country in our review (Table 1).

**Table 1:** Characteristics of included studies

Author/year	Country	Number of farms and animals	Sampling site at different distances	Control site	Location and type of wells
Swine					
Chee-Sanford et al. 2001	USA	2 farms with 1200–4000 animals	At different distances (up to 250 m) downstream of waste lagoons	Upstream of waste lagoons	Onsite monitoring wells
Koike et al. 2007	USA	2 farms with 1200–4000 animals	At different distances (up to 250 m) downstream of waste lagoons	Upstream of waste lagoons	Onsite monitoring wells
Koike et al. 2010	USA	2 farms with 1200–4000 animals	At different distances (up to 250 m) downstream of waste lagoons	Upstream of waste lagoons	Onsite monitoring wells
Mackie et al. 2006	USA	2 farms with 1200–4000 animals	At different distances (up to 250 m) downstream of waste lagoons	Upstream of waste lagoons	Onsite monitoring wells
Anderson and Sobsey 2006	USA	2 farms with 1500–5000 animals	On farms	Crop farms, “small herd of beef cattle” at one control site	Onsite monitoring wells
Sapkota et al. 2007	USA	1 farm with 3000 animals	400 m downgradient of farm	Upgradient of farm but near septic tank	Offsite private wells
Stine et al. 2007	USA	1 farm with 1200 animals/year	On farm	None	Onsite wells of unspecified use type
Hong et al. 2013	USA	3 farms with 2300–4000 animals	Adjacent to waste lagoon	Upgradient of waste lagoon	Onsite monitoring and facility wells
Casanova and Sobsey 2016	USA	County 1: 20 farms with 446,000 animals; County 2: 92 farms with 1.4 million animals	Wells in counties with swine farms but not on/adjacent to farm (distance from farms unspecified)	None	Offsite private wells and monitoring wells
He et al. 2016	China	3 farms with 5,600-16,600 animals/year	On farms	None (for groundwater samples)	Onsite wells of unspecified use type
Li et al. 2018	China	9 farms with unspecified number of animals	On farms and 2–3 km from farms	None	Onsite and offsite community wells
Huang et al. 2019	China	1 farm (joint with fish farm) with 12,000 animals	On farm	None	Onsite facility wells used for drinking water for farm workers
Gao et al. 2020	China	Unspecified number of farms with 2000 sows and 48,000 piglets per year	On farm and in village with swine farms (distance from farms unspecified)	Village with no swine farms	Onsite and offsite private wells and facility wells
Poultry					
Hubbard et al. 2020	USA	9 farms with > 1M chickens and > 42,000 turkeys	On turkey farms and adjacent (0.5–1.6 km) to chicken farms	Watershed with no poultry but receiving wastewater discharge and likely swine manure	Onsite and offsite private wells and facility
Alsalah et al., 2015	S. Arabia	1 farm with unspecified number of animals	< 20 km from farm	> 20 km from farm	Offsite wells of unspecified use type
Furtula et al. 2013	Canada	Unspecified number of farms and animals	Intensive poultry farming area (distance from farms unspecified)	Residential area	Offsite wells of unspecified use type
Wang et al. 2017	China	1 farm with unspecified number of animals	Within layer and 8–25 m away from layer	None	Onsite facility wells
Cattle					
Li et al. 2014	USA	2 farms with unspecified number of animals	On farms	None	Onsite monitoring wells
Li et al. 2015	USA	8 farms with unspecified number of animals; 1500 farms with 1.7 million cows in area	On farms, immediately downstream of manure-treated fields, waste lagoons and corrals, and < 2.4 km from dairy farms	> 2.4 km from farms (all offsite wells likely receiving manure and near septic tanks)	Onsite and offsite private wells and monitoring wells
Guo et al. 2021	USA	1 farm with 160 animals	Next to barn and in farmland receiving manure (1 km from barn)	None for groundwater	Onsite wells of unspecified use type
Mixed animals					
Economides et al. 2012	Cyprus	Unspecified number of farms and number of cattle, sheep, and goat	On farms	None	Onsite wells of unspecified use type
Blauth 2007	USA	11 swine and cattle farms with 2,800-7,444 swine and 0-250 cattle per farm	On farms	None (for groundwater)	Onsite facility wells
Gu et al. 2022	China	101 swine, 52 poultry, and 73 cattle farms with unspecified number of animals	On farms	None	Onsite wells of unspecified use type

### Animal husbandry operations

The animal husbandry operations included facilities for swine (13 studies), poultry (4 studies), cattle (3 studies), and multiple types of animals (3 studies). Of the studies with multiple types of animals; one focused on swine and cattle, one on swine, poultry, and cattle; and one on cattle, sheep, and goats. In high-income country studies, the number of animals that impacted study areas ranged from 1200 to 1.4 million swine, a “small herd” of less than 65 cows to 1.7 million cows, and poultry operations were often described more broadly as “areas of intensive poultry operation” with the maximum number of birds within a single study area equaling > 1 million birds (Table 1). In studies in China, swine operations ranged from 5600 to 50,000 animals within the study area; an animal count was not mentioned for studies that included poultry or cattle (Table 1). Only five studies in the USA included information on the antibiotic regimen used at the animal husbandry operation (Table 2). No studies in other countries provided information on the antibiotics used at the animal husbandry operations impacting the study site.

**Table 2:** Antibiotic regimen used in included studies (reported in five out of 23 studies)

Studies	Country	Type of animal	Antibiotics used
Chee-Sanford et al. 2001	USA	Swine	Chlortetracycline, tylosin
Blauth 2007	USA	Swine, cattle	Chlortetracycline, tylosin, tulathromycin, tetracycline, sulfamethoxazole, bisulfate, penicillin, gentamicin, ampicillin
Stine et al. 2007	USA	Swine	Chlortetracycline
Hong et al. 2013	USA	Swine	Chlortetracycline
Guo et al. 2021	USA	Cattle	Tulathromycin, ceftiofur, oxytetracycline

### Groundwater sampling

Groundwater samples in high-income country studies were collected from monitoring wells (9 studies), private wells (4 studies), facility wells (i.e., onsite wells serving the animal husbandry operations) (3 studies), and wells of unspecified use type (5 studies) (Table 1). Groundwater samples in studies in China were collected from facility wells (4 studies), private wells (1 study), community wells (1 study), and wells of unspecified use type (2 studies) (Table 1). Some studies sampled multiple types of wells. Well depth ranged from 3 to 76 m in high-income country studies and 6 to 143 m in studies in China.

Sampling locations included wells onsite of animal husbandry operations, offsite but proximate to (< 3 km) animal husbandry operations, and distant (> 3 km) from animal husbandry operations. We defined these categories based on common distances used within the studies. For example, some studies used the 2–3 km mark as a benchmark for the range of impact from animal husbandry operations, and larger distances were considered outside the range of impact (Li et al. 2015, 2018; Hubbard et al. 2020). Some studies sampled at multiple distances from the animal husbandry operations. Of the 17 high-income country studies, 13 studies sampled onsite wells, 3 studies sampled proximate wells, 2 studies sampled distant wells, and 2 studies sampled at an unspecified distance from an animal husbandry operation (Table 1). Of the 6 studies in China, all sampled onsite wells, and 1 study also sampled proximate wells, while 1 study also sampled at an unspecified distance from an animal husbandry operation (Table 1).

Twelve studies sampled groundwater at control sites (i.e., sites not expected to be impacted by animal husbandry operations) to serve as a comparison. Control sites included wells located onsite of an animal husbandry operation but upstream of waste lagoons (5 studies), upstream of animal husbandry operations (1 study), substantially distant (> 2.4 to > 20 km) from animal husbandry operations (2 studies) or in areas with no animal husbandry (e.g., crop farms, residential areas, villages with no farms) (4 studies) (Table 1). However, some of the control sites had animal influence from other sources. In one study focused on swine, the control site had a “small herd of beef cattle” (Anderson and Sobsey 2006). In another study focused on poultry, the control watershed received swine manure (Hubbard et al. 2020), and in a study focused on cattle, the control site received manure (Li et al. 2015). Some control sites were also at risk of contamination with human fecal waste from wastewater discharge (Hubbard et al. 2020) or septic tanks (Sapkota et al. 2007; Li et al. 2015). The remaining 11 studies did not sample groundwater at a control site.

### Assessment of AMR

Studies used a mix of culture-based methods to assess phenotypic resistance by enumerating ARB and molecular methods to assess genotypic resistance by detecting and/or enumerating ARGs; 10 studies used culture, 10 studies used molecular methods, and 3 studies used both (Table S2). Studies using culture methods primarily focused on *Escherichia coli* (*E. coli*), *Enterococcus*, and *Salmonella*. Of the studies using molecular methods, 10 extracted total DNA from groundwater samples and 3 isolated bacterial species and extracted DNA from isolates (Table S2). Most studies investigated tetracycline and sulfonamide resistance; these are among the most commonly used classes of antibiotics in animal husbandry (Van Boeckel et al. 2019). Studies also assessed resistance to clinically relevant antibiotics for human medicine, such as ciprofloxacin and erythromycin, and a small number of recent studies looked for resistance to beta-lactams (Gao et al. 2020; Hubbard et al. 2020) and carbapenem (Gu et al. 2022). Studies using molecular methods also investigated mobile genetic elements such as integrons (Hong et al. 2013; Li et al. 2015; He et al. 2016; Gao et al. 2020).

### Impact of animal husbandry operations on AMR in groundwater

#### Swine facilities

Of the nine studies focused on swine in high-income countries (all conducted in the USA), 67% (6/9) found evidence of groundwater contamination with ARB/ARGs resulting from swine operations (Table 3, Table S2). Contamination was detected in onsite monitoring wells (Chee-Sanford et al. 2001; Anderson and Sobsey 2006; Mackie et al. 2006; Koike et al. 2007, 2010) and in a private well located 400 m downgradient of a swine facility (Sapkota et al. 2007). Four of these studies focused on the same two swine farms and investigated tetracycline and macrolide-lincosamide- streptogramin B resistance genes; these studies found that monitoring wells located downstream and closer to waste lagoons were more likely to contain these ARGs than monitoring wells that were upstream of lagoons or downstream but further from lagoons (Chee-Sanford et al. 2001; Mackie et al. 2006; Koike et al. 2007, 2010). Another study found that 70% of *E. coli* isolated from onsite monitoring wells at two swine farms were resistant to antimicrobials (primarily tetracycline and chlortetracycline) compared to 18% of isolates from monitoring wells at two crop farms (Anderson and Sobsey 2006). The one study that found evidence of contamination in a private well found higher minimum inhibitory concentrations for multiple antibiotics and a higher prevalence of resistant *Enterococcus* isolates in a well located downgradient (400 m) vs. upgradient of the swine facility (Sapkota et al. 2007). However, the upgradient well was near a septic tank and had a higher prevalence of *Enterococcus* isolates resistant to erythromycin and vancomycin (the latter was not approved for veterinary use in the USA at the time) than the downgradient well.

**Table 3:** Impact from CAFOs on AMR in groundwater, by animal type, country income level, and well location and type

	Number of studies		Fraction of studies showing CAFO impact on AMR in groundwater								
	Total	With control site	Any well	By well location ^a^			By well type				
				Onsite	Offsite near ^b^	Offsite unspecified distance	Monitoring well	Private well	Facility well	Community well	Unspecified well type
High-income countries											
Swine	9	7	67% (6/9)	71% (5/7)	100% (1/1)	0% (0/1)	86% (6/7)	50% (1/2)	100% (1/1)	–	0% (0/1)
Poultry	3	3	33% (1/3)	0% (0/1)	0% (0/1)	100% (1/1)	–	0% (0/1)	0% (0/1)	–	50% (1/2)
Cattle	3	1	67% (2/3)	67% (2/3)	0% (0/1)	–	50% (1/2)	0% (0/1)	–	–	100% (1/1)
Mixed	2	0	100% (2/2)	100% (2/2)	–	–	–	–	100% (1/1)	–	100% (1/1)
Total	17	11	65% (11/17)	69% (9/13)	33% (1/3)	50% (1/2)	78% (7/9)	25% (1/4)	67% (2/3)	–	60% (3/5)
Low- and middle-income countries^c^											
Swine	4	1	100% (4/4)	100% (4/4)	100% (1/1)	0% (0/1)	–	100% (1/1)	100% (3/3)	100% (1/1)	100% (1/1)
Poultry	1	0	100% (1/1)	100% (1/1)	–	–	–	–	–	–	–
Cattle	0	–	–	–	–	–	–	–	–	–	–
Mixed	1	0	100% (1/1)	100% (1/1)	–	–	–	–	100% (1/1)	–	100% (1/1)
Total	6	1	100% (6/6)	100% (6/6)	100% (1/1)	0% (0/1)	–	100% (1/1)	100% (4/4)	100% (1/1)	100% (2/2)

^a^Some studies sampled at multiple distances from animal husbandry operations (e.g., both onsite and offsite)

^b^Within 3 km of animal husbandry operation

^c^China (upper middle income) was the only country in this category

Three studies in the USA found no conclusive evidence of ARB/ARG contamination from swine operations (Table S2). One study detected no tetracycline-resistant bacteria in an onsite well of unspecified use type (Stine et al. 2007), and another study found no resistance to multiple antibiotics in *Enterococcus* isolates from monitoring wells and private wells in counties with swine farms but not located directly on or adjacent to the farms (Casanova and Sobsey 2016). Finally, one study on three swine farms detected ARGs in onsite monitoring wells located downgradient but not upgradient of waste lagoons, but this study also detected ARGs in a facility well located upgradient of lagoons (Hong et al. 2013).

Of the four studies focused on swine facilities in China, all found some evidence of contamination with ARB/ARGs associated with swine facilities (Table 3, Table S2). One study investigated facility wells on swine farms and private wells in villages with and without swine farms; the absolute abundance of ARGs was highest in the onsite facility wells and similar between villages with vs. without swine farms, while the relative abundance of ARGs was similar at all sites (Gao et al. 2020). Conversely, another study that focused on facility wells on swine farms and private wells 2–3 km away from farms found that the absolute abundance of ARGs was similar in onsite and offsite wells, while the relative abundance was higher in the onsite wells (Li et al. 2018). Two additional studies detected a range of ARGs in onsite wells, but these studies had no control site for comparison (He et al. 2016; Huang et al. 2019). The most commonly detected ARGs across the studies in China included tetracycline and sulfonamide resistance genes.

#### Poultry facilities

Of the three studies focused on poultry in high-income countries (USA, Saudi Arabia, Canada), 33% (1/3) found evidence of groundwater contamination with ARB resulting from poultry operations (Table 3, Table S2). In this study, 100% of *Enterococcus* isolates from wells in an area of intensive poultry farming in Canada were resistant to 2+ antibiotics, while no *Enterococcus* was detected in the control well in a residential area (Furtula et al. 2013). Conversely, the US study focused on onsite facility wells on turkey farms, private wells adjacent (0.5–1.6 km) to chicken farms, and a private well in a watershed with no poultry farms and found that, while 77% of samples had 1+ ARG, and contamination was most frequent in the control watershed with no poultry farms; the authors noted that this watershed received wastewater discharge and likely swine manure (Hubbard et al. 2020). The study in Saudi Arabia investigated wells < 20 km vs. > 20 km from a poultry farm and did not detect ARB in any well (Alsalah et al. 2015). One study focused on poultry farms in China and found evidence of groundwater contamination with ARB (Table 3, Table S2). This study tested facility wells inside and immediately adjacent (8–25 m) to the layer; no bacteria were isolated from wells 14–25 m away from the layer, while isolates from the other wells were resistant to several antibiotics (Wang et al. 2017).

#### Cattle facilities

Of the three studies focused on cattle (all conducted in the USA), 67% (2/3) found evidence of contamination of groundwater with ARB/ARGs within the study area, including monitoring wells and wells of unspecified use type located on cattle farms (Table 3, Table S2). One of these studies found that 25% of *E. coli* isolates from two monitoring wells onsite of dairy farms showed resistance to ceftriaxone and tetracycline and intermediate resistance to chloramphenicol (Li et al. 2014). The other study only sampled two wells: one was located next to a barn that housed cattle and no ARGs were detected in this well, the other was located 1 km away from the barn on a farm property that received applied manure, and multidrug resistance genes were detected in this well (Guo et al. 2021). Neither study had a control site for comparison. The third study with no conclusive evidence sampled 46 monitoring wells and 5 private wells onsite on eight dairy farms, as well as 200 private or small community wells located < 2.4 km vs. > 2.4 km from the dairy farms (Li et al. 2015). The study found that 64% of *E. coli* isolates and 86% of *Enterococcus* isolates from the wells were resistant to 3+ antibiotics, but the prevalence of resistance was not different between onsite monitoring vs. private wells or between offsite wells located < 2.4 km vs. > 2.4 km from the dairy farms. However, in this study, all offsite wells were near croplands that likely received manure and were also near septic tanks.

#### Multiple types of facilities

Of the two studies in high-income countries (USA, Cyprus) that focused on multiple types of animals, both found contamination of groundwater with ARB/ARGs associated with the facilities (Table 3, Table S2). The US study found that, among onsite facility wells at 11 farms housing swine and cattle, 100% of *E. coli* isolates were resistant to tetracycline and 80% of *Enterococcus* isolates were resistant to three antibiotics (Blauth 2007). The study in Cyprus sampled wells of unspecified use type located onsite at cattle, sheep, and goat farms and found that 48% of *Salmonella* isolates and 30% of *E. coli* isolates were resistant to at least one antibiotic (Economides et al. 2012). One study focused on swine, poultry, and cattle farms in China and found evidence of groundwater contamination with ARB (Table 3, Table S2). This study sampled a total of 208 private and facility wells located onsite of the farms and detected carbapenem-resistant Enterobacteriaceae (CRE) in 5% of wells; the prevalence of CRE was significantly higher on poultry farms than on swine or cattle farms and similar on swine and cattle farms (Gu et al. 2022). None of the three studies used a control site or sampled offsite of the animal facilities they were investigating.

### Effect of well type and sampling distance from animal husbandry operations

AMR contamination was most commonly found in facility wells serving animal husbandry operations (100% in China, 67% in high-income countries) and in monitoring wells (78% in high-income countries) (Table 3). While studies collected samples at varying distances from animal husbandry operations, contamination was most commonly detected onsite of the operations. In high-income countries, evidence of AMR in groundwater associated with animal husbandry operations was found in 69% (9/13) of studies that sampled wells onsite of the operations, 33% (1/3) of studies that sampled offsite within 3 km of the operations, and 50% (1/2) of studies that sampled offsite at an unspecified distance (Table 3). In studies in China, AMR associated with animal husbandry operations was detected in 100% (6/6) of studies that sampled onsite and in the one study that sampled within 3 km of the operations (Table 3).

Only three studies found groundwater contamination resulting from these operations at offsite locations, including a private well 400 m downgradient of a swine farm with 3000 animals in the USA (Sapkota et al. 2007), private wells located within 2–3 km of swine farms (number of animals unspecified) in China (Li et al. 2018), and wells located in an area with intensive poultry farming (distance from farms and number of animals unspecified) in Canada (Furtula et al. 2013). Two of these studies had a control site (private well upgradient of the swine farm (Sapkota et al. 2007), well in a residential area (Furtula et al. 2013)), which better proves a measurable impact on groundwater from an animal husbandry operation than a positive result without a control site. However, the control site in one study was impacted by a septic tank (Sapkota et al. 2007). All other studies that detected ARB/ARGs in groundwater sampled onsite of animal husbandry operations.

### Effect of onsite antibiotic use

Of the five studies that included information on the antibiotic regimen used on the facility, two found resistance to the specific antibiotics used (tetracyclines, macrolides) more commonly at sites impacted by the swine facilities than control sites (Chee-Sanford et al. 2001; Hong et al. 2013). In another study where tetracyclines were used at the facility at subtherapeutic doses and an array of other antibiotics were used therapeutically, 100% of *E. coli* isolates from onsite facility wells were resistant to tetracycline but not to other antibiotics, while 80% of *Enterococcus* isolates from the same wells were resistant to 3 antibiotics; this study did not sample groundwater at a control site (Blauth 2007). In contrast, one study did not detect any tetracycline-resistant bacteria in a well on a swine farm despite the use of chlortetracycline-containing feed (Stine et al. 2007). In another study where ceftiofur, tulathromycin, and oxytetracycline were used therapeutically on dairy farms, qPCR for 113 ARGs and 21 mobile genetic elements detected no ARGs in a well next to barns, and only the multidrug resistance genes mexF and qacEΔ1 were detected in a well within adjacent farmlands receiving manure (Guo et al. 2021). Additionally, one study tested *Enterococcus* isolates for resistance to an antibiotic (vancomycin) not approved for veterinary use at the time; minimum inhibitory concentrations were higher for several antibiotics, but not vancomycin, in a private well downgradient of the swine facility compared to an upgradient private well (Sapkota et al. 2007).

### Effects of climatic conditions and precipitation

Only seven studies included information about either the site-specific climatic conditions or the precipitation and temperatures that occurred during the study (Anderson and Sobsey 2006; Blauth 2007; Li et al. 2014, 2015; He et al. 2016; Huang et al. 2019; Gu et al. 2022). One study found contamination of groundwater with ARB during an approximately 2-year sampling period even though an especially dry climate occurred during the last half of the sampling period (Anderson and Sobsey 2006). Three studies purposefully sampled during different seasons or weather conditions (Blauth 2007; Li et al. 2015; Huang et al. 2019), although several other studies sampled in multiple seasons and were likely impacted by varying precipitation and temperature as well (Mackie et al. 2006; Koike et al. 2007, 2010). One study found that the abundance of ARGs was one order of magnitude higher during the wet vs. dry season (Huang et al. 2019), and another study found a higher prevalence of resistance during the sampling round with cool and wet weather (Li et al. 2014).

## Discussion

### Summary of findings

Of the 23 studies included in our review, the majority were conducted in high-income countries (mostly the USA), and over half of the studies focused on swine facilities. Of the studies in high-income countries, 65% (11/17) found evidence of ARB/ARG contamination associated with the animal facilities (6/9 for swine, 1/3 for poultry, 2/3 for cattle, 2/2 for multiple animal types). Of the studies in China, the only upper-middle-income country where studies in our review were conducted, 100% (6/6) found some evidence of contamination (4/4 for swine, 1/1 for poultry, 1/1 for multiple animal types). Of the studies demonstrating impact, only 7 out of 11 studies in high-income countries and 1 out of 6 studies in China sampled a control site, but some of the control sites received contamination from animal or human fecal sources or both. Among the five studies that reported the antibiotics used at the investigated operations, resistance to those specific antibiotics was detected in three of the studies.

### Implications for private drinking water wells

The studies in our review mostly focused on monitoring wells and wells serving the needs of the facilities, while only four out of 23 studies sampled private drinking water wells. Studies mostly sampled wells located onsite at animal husbandry operations; only 10 studies sampled offsite wells (5 located < 3 km, 2 located > 3 km, and 3 located at an unspecified distance from the facilities). Evidence of contamination with ARB/ARGs was mostly detected in onsite wells. The largest distance where contamination was detected in an offsite private well was 2–3 km away from swine farms in China. In the USA, contamination was detected in a private well 400 m downgradient of a swine farm. These findings indicate some risk of exposure to ARB/ARGs through drinking water from private wells that are located on the same property as, or in the vicinity of, animal husbandry operations. Private well users in rural areas are vulnerable to drinking water contaminants since water from these wells is often not tested or treated (Gibson and Pieper 2017). Studies should further assess the presence of ARB/ARGs in private wells at varying distances downgradient of animal husbandry operations to better determine the risk of waterborne exposure to AMR associated with these operations. Rural wells are also vulnerable to human fecal contamination from non-sewered sanitation systems such as septic tanks. Therefore, it is important that future studies sample groundwater from control sites to be able to isolate contamination stemming from animal husbandry operations. Control sites should be carefully selected to avoid unaccounted-for contamination from animal or human fecal sources to rule out outside sources of ARB/ARGs and antibiotics and more definitively attribute AMR contamination to animal husbandry operations.

### Research needs in the context of climate change

Our findings indicate that, while contamination of groundwater from animal husbandry operations can occur under both rainy and dry conditions, there is some evidence that contamination is more pronounced during periods of increased rainfall. However, few studies in our review reported effects stratified by weather conditions. Widespread fecal contamination from animal husbandry operations has been previously reported in waterbodies in the wake of extreme weather events and hurricanes through breaching and flooding of lagoons (Wing et al. 2002; Harris et al. 2021). Rainfall can lead to increased runoff from fields where manure from animal husbandry operations has been applied (Thurston-Enriquez et al. 2005). Wetter soil conditions during and after rainfall can also hydraulically enhance contaminant transport in the subsurface (Buckerfield et al. 2019). Episodes of heavy rainfall are expected to become more frequent due to global warming. Future research should investigate the effect of rainfall on the spread of AMR from animal husbandry operations into surrounding groundwater aquifers by purposefully sampling after rainfall events of different intensities.

### Need for data from low- and middle-income countries

Rates of AMR in food animals differ between high- and low-income countries. Loose regulations on veterinary use of antimicrobials leading to their increased use, along with poorly nourished animals and less stringent biosecurity in the facilities, could lead to higher rates of resistance in food animals in low-income countries, although it is also possible that access to veterinary antimicrobials is limited, particularly in rural low-income country settings (Van Boeckel et al. 2019). Our findings indicate that groundwater contamination with ARB/ARGs associated with animal feeding operations may be more prevalent but is also less well studied in low- and middle-income countries. Only six of the 23 studies in our review were conducted in an upper-middle-income country; these studies were exclusively conducted in China and all suggested evidence of AMR in groundwater associated with animal feeding operations. China, a leading AMR hotspot, is one of the largest global consumers of antimicrobials in food animals and has been intensifying its meat production operations (Van Boeckel et al. 2015, 2019). In Asia, Africa, and South America, meat production has grown by 68%, 64%, and 40%, respectively, between 2000 and 2019 due to increasing demand for high-protein diets, while growth in high-income countries has plateaued (Van Boeckel et al. 2019). Studies on waterborne exposure to AMR from animal feeding operations are needed in other existing and emerging hotspots of resistance in food animals in low- and middle-income countries, such as India, Brazil, and Kenya (Van Boeckel et al. 2019).

### Human health risk from AMR from animal husbandry operations

The ultimate human health risk from waterborne exposure to AMR from animal feeding operations is not well understood. Whole genome sequencing studies at animal farms in the USA have found overlapping genotypic resistance profiles between the animals, farmers, and the farm environment, indicating exchange between these reservoirs and hosts, but the directionality of transmission is not clear (Pornsukarom et al. 2018). ARB have been detected in the nasal passages of US animal farm workers (Nadimpalli et al. 2015; Hatcher et al. 2017). A recent review has found that exposure to domestic animals in backyard farms is associated with AMR carriage among household members in low- and middle-income countries despite rates of resistance being lower among animals raised in backyard farms than in small-scale or industrial facilities (Swarthout et al. 2022). The review also identified water, soil, and animal products as potential transmission pathways (Swarthout et al. 2022). While the risk of human colonization or infections with ARB from waterborne exposure to these organisms remains unquantified, a study in Canada found that drinking water from private wells contaminated with antimicrobial-resistant *E. coli* was associated with a 26% increase in the risk of gut colonization with this organism (Coleman et al. 2012).

### Measures to reduce risk of AMR from animal husbandry operations

Two regulatory measures can serve to limit AMR risks associated with animal feeding operations: reduced antimicrobial use in food animals and proper waste management practices. A United Nations investigation has found that inappropriate veterinary antimicrobial use is the leading cause of AMR in food animals and contributes to increased carriage of AMR in humans (Van Boeckel et al. 2017). A systematic review found that restricting the veterinary use of antimicrobials reduced ARB prevalence by 15% in food animals and 24% among humans (Tang et al. 2017). Such restrictions could be highly effective in countries where meat production is intensifying. Poor management of animal waste is also directly linked to increased risk of dissemination of AMR from animal feeding operations (Swarthout et al. 2022). One study in the USA found that stagnant waste lagoons at cattle farms contained twice as many ARGs as agitated lagoons and eight times as many ARGs as cattle fecal samples (Guo et al. 2021), corroborating the role of waste management practices in the proliferation of AMR. Improved management of animal waste to reduce contamination of water sources with AMR and/or effective point-of-use water treatment is particularly important in areas where residents obtain drinking water from private wells.

### Limitations

Although our systematic review effectively demonstrated that animal husbandry operations present a risk to groundwater quality with respect to AMR, there are a few limitations to consider. The studies eligible for inclusion in our review were mostly conducted in the USA and China, and studies were lacking from low-income countries with intensifying animal production and high rates of AMR. Only five studies included information about the antibiotic regimen used at the animal husbandry operation, which can help assess causality by comparing whether any detected AMR profiles overlap with the antimicrobials used at the site. While most studies reported well depth, many studies did not include information on the depth of the water table or the soil type, which are important determinants of the risk of contaminant infiltration from the surface. Few studies reported effects stratified by weather conditions to allow assessing the impacts of rainfall on the dissemination of AMR from animal husbandry operations. Additionally, we only included studies that sampled groundwater with respect to proximity to an active animal feeding operation and excluded studies that solely focused on manure application without reference to a specific facility. Liquid waste from swine operations is typically disposed of in the immediate vicinity of the facilities, while dry waste from poultry operations can be transported up to 15 km before being applied to fields (Miralha et al. 2021). Most studies in our review sampled groundwater within 2–3 km from facilities; while this captures most instances of liquid manure application, we may have not captured groundwater risks associated with the application of dry manure at more distant locations. We have also not investigated the role of surface waters or non-waterborne routes through which AMR can disseminate from animal operations, such as soil, produce, and animal products and direct contact with farm animals and/or their feces (Silbergeld et al. 2008; Graham et al. 2019). Additionally, because the included studies investigated diverse outcomes (different bacteria, antibiotic resistance profiles, and resistance genes) and reported a range of outcome measures (prevalence, absolute abundance, relative abundance), we were not able to quantitatively pool outcome measures in a meta-analysis.

## Conclusions

Overall, our findings indicate that animal husbandry operations result in groundwater contamination with ARB/ARGs, although most evidence is available from monitoring wells onsite of the operations. Future studies should sample private wells at varying distances from animal husbandry operations under various weather conditions to better characterize the risk of waterborne exposure to AMR from animal husbandry operations in the context of climate change. Studies should sample appropriately selected control sites to rule out other sources of human and animal fecal contamination and better isolate the impact of animal husbandry operations. Additional groundwater quality studies are needed in existing and emerging AMR hotspots for food animals in low- and middle-income countries with intensifying meat production.

### Supplementary information

ESM 1 (PDF 784 kb)
