# Supplementary material for: Do animal husbandry operations contaminate groundwater sources with antimicrobial resistance: systematic review
Source: Environ Sci Pollut Res Int. 2024 Feb 6;31(11):16164–76. doi: 10.1007/s11356-024-31899-w (PMC10894137; doi:10.1007/s11356-024-31899-w)
Supplement: Supplementary file 1 — (PDF 784 kb) [file 11356_2024_31899_MOESM1_ESM.pdf]

Supplemental Information

Do animal husbandry operations contaminate groundwater sources with antimicrobial resistance: Systematic review

Text S1. Database search strings

PubMed

((("animal husbandry") OR ("animal agriculture") OR ("concentrated animal feeding operation*") OR ("confined animal feedling operation*") OR ("confinement facilit*") OR (CAFO) OR ("feeding operation*") OR ("feeding facilit*") OR ("livestock production") OR ("animal operation*") OR ("animal production") OR ("livestock operation*") OR ("livestock farm*") OR ("pig farm*") OR ("hog farm*") OR ("swine farm*") OR ("pig production*") OR ("hog production*") OR ("swine production*") OR ("pig operation*") OR ("hog operation*") OR ("swine operation*") OR ("chicken farm*") OR ("poultry farm*") OR ("chicken production*") OR ("poultry production*") OR ("chicken operation*") OR ("poultry operation*") OR ("broiler farm*") OR ("broiler production*") OR ("broiler operation*") OR ("cattle operation*") OR ("dairy operation*") OR ("cow farm*") OR ("cattle farm*") OR ("dairy farm*") OR ("cattle production") OR ("dairy production") OR ("cattle ranch*") OR ("cow production*") OR ("waste lagoon*") OR (lagoon) OR ("anaerobic lagoon*") OR ("poultry indust*") OR ("swine industr*") OR ("cattle industr*") OR ("poultry barn*") OR ("hen farm*") OR (feedlot)) AND (("antimicrobial resistan*") OR ("antibiotic resistan*") OR ("resistance gene*") OR ("multi drug resistan*") OR ("multi resistan*") OR ("drug resistan*") OR ("pan resistan*"))) AND ((groundwater) OR ("well water*") OR (aquifer) OR ("monitoring well*") OR ("domestic well*") OR ("private well*") OR (borehole) OR (tubewell) OR ("tube well") OR ("water well") OR ("water suppl*") OR ("ground water") OR ("residential well*"))

Web of Science

((ALL=("animal husbandry")) OR ALL=("animal agriculture")) OR ALL=("concentrated animal feeding operation*")) OR ALL=("confined animal feeding operation*")) OR ALL=("confinement facilit*")) OR ALL=(CAFO)) OR ALL=("feeding operation*")) OR ALL=("feeding facilit*")) OR ALL=("livestock production")) OR ALL=("animal operation*")) OR ALL=("animal production")) OR ALL=("livestock operation*")) OR ALL=("livestock farm*")) OR ALL=("pig farm*")) OR ALL=("hog farm*")) OR ALL=("swine farm*")) OR ALL=("pig production*")) OR ALL=("hog production*")) OR ALL=("swine production*")) OR ALL=("pig operation*")) OR ALL=("hog operation*")) OR ALL=("swine operation*")) OR ALL=("chicken farm*")) OR ALL=("poultry farm*")) OR ALL=("chicken production*")) OR ALL=("poultry production*")) OR ALL=("chicken operation*")) OR ALL=("poultry operation*")) OR ALL=("broiler farm*")) OR ALL=("broiler production*")) OR ALL=("broiler operation*")) OR ALL=("cattle operation*")) OR ALL=("dairy operation*")) OR ALL=("cow farm*")) OR ALL=("cattle farm*")) OR ALL=("dairy farm*")) OR ALL=("cattle production")) OR ALL=("dairy production")) OR ALL=("cattle ranch*")) OR ALL=("cow production*")) OR ALL=("waste lagoon*")) OR ALL=(lagoon)) OR ALL=("anaerobic lagoon*")) OR ALL=("poultry indust*")) OR ALL=("swine

industr*")) OR ALL=("cattle industr*")) OR ALL=("poultry barn*")) OR ALL=("hen farm*")) OR ALL=(feedlot)

((((((ALL=("antimicrobial resistan*")) OR ALL=("antibiotic resistan*")) OR ALL=("resistance gene*")) OR ALL=("multi drug resistan*")) OR ALL=("multi resistan*")) OR ALL=("drug resistan*")) OR ALL=("pan resistan*")

(((((((((((ALL=(groundwater)) OR ALL=("well water*")) OR ALL=(aquifer)) OR ALL=("monitoring well*")) OR ALL=("domestic well*")) OR ALL=("private well*")) OR ALL=(borehole)) OR ALL=(tubewell)) OR ALL=("tube well*")) OR ALL=("water well*")) OR ALL=("ground water")) OR ALL=("residential well*")

CAB Direct & NCSU Agricultural and Environmental Science

(groundwater OR "well water*" OR aquifer OR "monitoring well*" OR "domestic well*" OR "private well*" OR borehole OR tubewell OR "tube well*" OR "water well*" OR "ground water" OR "residential well*") AND ("antimicrobial resistan*" OR "antibiotic resistan*" OR "resistance gene*" OR "multi drug resistan*" OR "multi resistan*" OR "drug resistan*" OR "pan resistan*") AND ("animal husbandry" OR "animal agriculture" OR "concentrated animal feeding operation*" OR "confined animal feeding operation*" OR "confinement facilit*" OR CAFO OR "feeding operation*" OR "feeding facilit*" OR "livestock production" OR "animal operation*" OR "animal production" OR "livestock operation*" OR "livestock farm*" OR "pig farm*" OR "hog farm*" OR "swine farm*" OR "pig production*" OR "hog production*" OR "swine production*" OR "pig operation*" OR "hog operation*" OR "swine operation*" OR "chicken farm*" OR "poultry farm*" OR "chicken production*" OR "poultry production*" OR "chicken operation*" OR "poultry operation*" OR "broiler farm*" OR "broiler production*" OR "broiler operation*" OR "cattle operation*" OR "dairy operation*" OR "cow farm*" OR "cattle farm*" OR "dairy farm*" OR "cattle production" OR "dairy production" OR "cattle ranch*" OR "cow production*" OR "waste lagoon*" OR lagoon OR "anaerobic lagoon*" OR "poultry indust*" OR "swine industr*" OR "cattle industr*" OR "poultry barn*" OR "hen farm*" OR feedlot)

Table S1. PRISMA checklist

Section/Topic	#	Checklist Item	Page #
**TITLE**			
Title	1	Identify the report as a systematic review, meta-analysis, or both.	Title
**ABSTRACT**			
Structured summary	2	Provide a structured summary including, as applicable: background; objectives; data sources; study eligibility criteria, participants, and interventions; study appraisal and synthesis methods; results; limitations; conclusions and implications of key findings; systematic review registration number.	Abstract
**INTRODUCTION**			
Rationale	3	Describe the rationale for the review in the context of what is already known.	Introduction, paragraph 4
Objectives	4	Provide an explicit statement of questions being addressed with reference to participants, interventions, comparisons, outcomes, and study design	Introduction, paragraph 4
**METHODS**			
Protocol and registration	5	Indicate if a review protocol exists, if and where it can be accessed (e.g., Web address), and, if available, provide registration information including registration number.	N/A
Eligibility criteria	6	Specify study characteristics (e.g., PICOS, length of follow-up) and report characteristics (e.g., years considered, language, publication status) used as criteria for eligibility, giving rationale.	Materials and methods; Criteria for inclusion and exclusion
Information sources	7	Describe all information sources (e.g., databases with dates of coverage, contact with study authors to identify additional studies) in the search and date last searched.	Materials and methods; Literature search
Search	8	Present full electronic search strategy for at least one database, including any limits used, such that it could be repeated.	Text S1
Study selection	9	State the process for selecting studies (i.e., screening, eligibility, included in systematic review, and, if applicable, included in the meta-analysis).	Materials and methods; Literature search
Data collection process	10	Describe method of data extraction from reports (e.g., piloted forms, independently, in duplicate) and any processes for obtaining and confirming data from investigators.	Materials and methods; Data extraction and synthesis
Data items	11	List and define all variables for which data were sought (e.g., PICOS, funding sources) and any assumptions and simplifications made.	Materials and methods; Data extraction and

			synthesis
Risk of bias in individual studies	12	Describe methods used for assessing risk of bias of individual studies (including specification of whether this was done at the study or outcome level), and how this information is to be used in any data synthesis.	N/A
Summary measures	13	State the principal summary measures (e.g., risk ratio, difference in means).	Materials and methods; Data extraction and synthesis
Synthesis of results	14	Describe the methods of handling data and combining results of studies, if done, including measures of consistency (e.g., I2) for each meta-analysis.	Materials and methods; Data extraction and synthesis
Risk of bias across studies	15	Specify any assessment of risk of bias that may affect the cumulative evidence (e.g., publication bias, selective reporting within studies).	N/A
Additional analyses	16	Describe methods of additional analyses (e.g., sensitivity or subgroup analyses, meta-regression), if done, indicating which were pre-specified.	N/A
**RESULTS**			
Study selection	17	Give numbers of studies screened, assessed for eligibility, and included in the review, with reasons for exclusions at each stage, ideally with a flow diagram.	Results; Literature search and screening
Study characteristics	18	For each study, present characteristics for which data were extracted (e.g., study size, PICOS, follow-up period) and provide the citations.	Results; Study characteristics; Table 1
Risk of bias within studies	19	Present data on risk of bias of each study and, if available, any outcome-level assessment (see Item 12).	N/A
Results of individual studies	20	For all outcomes considered (benefits or harms), present, for each study: (a) simple summary data for each intervention group and (b) effect estimates and confidence intervals, ideally with a forest plot.	Results, Table 3, Table S2
Synthesis of results	21	Present results of each meta-analysis done, including confidence intervals and measures of consistency.	Results
Risk of bias across studies	22	Present results of any assessment of risk of bias across studies	N/A
Additional analysis	23	Give results of additional analyses, if done (e.g., sensitivity or subgroup analyses, meta-regression	N/A
**DISCUSSION**			
Summary of evidence	24	Summarize the main findings including the strength of evidence for each main outcome; consider their relevance to key groups (e.g., health care providers, users, and policy makers).	Discussion; Summary of findings
Limitations	25	Discuss limitations at study and outcome level (e.g., risk of bias), and at	Discussion;

		review level (e.g., incomplete retrieval of identified research, reporting bias).	Limitations
Conclusions	26	Provide a general interpretation of the results in the context of other evidence, and implications for future research.	Discussion; Implications of findings; Conclusions
**FUNDING**			
Funding	27	Describe sources of funding for the systematic review and other support (e.g., supply of data); role of funders for the systematic review.	N/A

Table S2. Characteristics and findings of studies included in review

Study	Setting	Sampling information	ARB/ARGs investigated	Findings
**Swine**				
**High-income countries**				
Chee-Sanford et al. 2001	USA<br>2 farms<br>1,200-4,000 animals	Onsite monitoring wells<br>18 wells downstream of waste lagoons, at different distance (up to 250 m) from lagoons<br>Control site: 2 wells upstream of waste lagoons	Bacterial isolates on plates with tetracycline<br>DNA extracted from tetracycline-resistant bacterial isolates and from groundwater<br>PCR for tetracycline (tetO, tetQ, tetW, tetM, tetB/P, tetS, otrA, tetT) resistance genes	Downstream wells predominantly had tetQ, tetW, tetM, tetT, tetO<br>ARGs detected in wells up to 250 m downstream of lagoons<br>Upstream well had tetT in Farm 1 and no ARGs in Farm 2
Koike et al. 2007	Same as above	Onsite monitoring wells<br>18 wells downstream of waste lagoons, at different distance (up to 250 m) from lagoons<br>Control site: 4 wells upstream of waste lagoons	DNA extracted from groundwater<br>PCR and qPCR for tetracycline (tetM, tetO, tetQ, tetW, tetC, tetH, tetZ) resistance genes	All ARGs consistently detected in 5 downstream wells close to lagoon at Farm 1; downstream wells further from lagoon and upstream wells had less frequent ARG detection<br>At Farm 1, wells had higher relative abundance of tetC than lagoons. At Farm 2, ARG abundance was below limit of quantification.
Koike et al. 2010	Same as above	Same as above	DNA extracted from groundwater<br>PCR for macrolide-lincosamide- streptogramin B (tlrB, tlrD, ermA, ermB, ermC, ermF, ermG, ermQ) resistance genes<br>qPCR for ermA, ermB, ermC, ermF	ermA, ermB, ermC, ermF detected in multiple wells at both farms; tlrD detected once in one well; tlrB not detected<br>Downstream wells close to lagoon at Farm 1 more likely to contain ARGs than other wells; ermB, ermC almost always detected in these wells; ermA, ermF not detected in any other wells<br>Abundance of erm genes below limit of quantification
Mackie et al. 2006	Same as above	Same as above	DNA extracted from groundwater<br>PCR for tetracycline (tetM, tetO, tetQ, tetW) resistance genes<br>qPCR for tetM and tetQ	At Farm 1, all ARGs detected in wells at some point with no clear temporal trends. At Farm 2, ARGs varied from mostly non-detect to mostly detected between rounds<br>Downstream wells further from lagoon and upstream wells had less frequent ARG detection
Anderson et al. 2006	USA<br>2 farms<br>1,500-5,000 animals	Onsite monitoring wells<br>37 wells on swine farms<br>Control site: 13 wells on two crop farms, "small herd of beef cattle" at one control site	*E. coli* isolates tested for susceptibility to streptomycin, vancomycin, chlortetracycline, tetracycline, trimethoprim, sulfamethoxazole, chloramphenicol, tiamulin, erythromycin, enrofloxacin, ciprofloxacin, neomycin, gentamicin, ampicillin, florfenicol, tylosin base, clindamycin	70% (63/90) of isolates resistant at swine farm wells, predominantly resistant to tetracycline, chlortetracycline<br>18% (2/11) of isolates resistant at crop farm wells
Sapkota et al. 2007	USA<br>1 farm<br>3,000 animals	Offsite private wells<br>1 domestic well 400 m downgradient of farm (no longer used for drinking)<br>Control site: 1 domestic well upgradient of farm but near septic tank	*Enterococcus* isolates tested for susceptibility to erythromycin, clindamycin, tetracycline, virginiamycin (streptogramin A and B combination), vancomycin – the latter not approved for veterinary use in the US	MICs for erythromycin, tetracycline, clindamycin and virginiamycin higher in downgradient well than upgradient well but not for vancomycin<br>19-100% of isolates resistant downgradient, 3-67% of isolates resistant upgradient<br>Resistant *Enterococcus* more common down vs. upgradient for clindamycin ($p<0.001$) and tetracycline ($p=0.07$) but less common down vs. upgradient for erythromycin ($p<0.001$) and vancomycin ($p=0.15$)
Stine et al.	USA	Onsite wells of unspecified use type	*E. coli* tested for susceptibility to tetracycline,	No tetracycline-resistant bacteria isolated from well

2007	1 farm<br>1,200 animals per year	1 well on farm<br>Control site: None	chloramphenicol, ciprofloxacin. *Enterococcus* tested for susceptibility to quinupristin/dalfopristin, vancomycin<br>DNA extracted from bacteria isolated from plates with tetracycline<br>PCR for tetracycline (tetA, tetB, tetC, tetE, tetH, tetL, tetM, tetS, tetT, rumB) resistance genes	
Hong et al. 2013	USA<br>3 farms<br>2,300-4,000 animals	Onsite monitoring and facility wells<br>3 monitoring wells adjacent to waste lagoon<br>Control site: 1 facility well and 1 monitoring well upgradient of lagoon	DNA extracted from groundwater<br>qPCR for tetracycline (tetQ, tetZ) resistance genes and integrons (intI1, intI2)	ARGs detected in all monitoring wells except for well upgradient of lagoon and in facility well upgradient of lagoon (deep well sampled from tap)<br>Varying abundance for different ARGs, wells and sampling rounds
Casanova and Sobsey 2016	USA<br>County 1: 20 farms, 446,000 animals<br>County 2: 92 farms 1.4 million animals	Offsite private wells and monitoring wells<br>64 wells in counties with swine farms but not on/adjacent to farm (distance from farms unspecified)<br>Control site: None	*Salmonella, E. coli, Enterococcus* isolated from groundwater<br>*Salmonella* and *E. coli* tested for susceptibility to trimethoprim, sulfamethoxazole, chloramphenicol, gentamicin, ampicillin, ciprofloxacin, ceftriaxone<br>*Enterococci* tested for susceptibility to vancomycin, streptomycin, chloramphenicol, gentamicin, ampicillin	No *Salmonella* or *E. coli* in wells, *Enterococcus* in 10% (3/30) of wells in one county<br>No resistance in *Enterococcus* from wells
**Low-and middle-income countries**				
He et al. 2016	China<br>3 farms<br>5,600-16,600 animals/year	Onsite wells of unspecified use type<br>3 wells on 3 swine farms<br>Control site: None (for groundwater samples)	DNA extracted from groundwater<br>qPCR for for sulfonamide (sul1, sul2, sul3), tetracycline (tetA, tetG, tetH, tetM, tetO, tetQ, tetS, tetW, tetB/P, tetT, tetX), chloramphenicol (cmlA, floR, fexA, fexB, cfr), macrolide– lincosamide–streptogramin B (ermB, ermC, ermE) resistance genes and integrons (intl1, intl2)	91% (+/-11%) of targeted ARGs detected in wells<br>Most common ARGs in wells: tetA, ermE, tetH, sul2
Li et al. 2018	China<br>9 farms<br>Unspecified number of animals	Onsite and offsite community wells<br>9 facility wells on farm<br>9 domestic wells 2-3 km from farm<br>Control site: None	DNA extracted from groundwater<br>qPCR for quinolone (qnrA), sulfonamide (sul1, sul2), tetracycline (tetG, tetM, tetO) resistance genes and class I integron (intl1)	tetG, tetM, tetO detected in all wells; qnrA, sul1, and sul2 only detected in on-site wells<br>Absolute abundance of ARGs similar in on-site and off-site wells, relative abundance lower in off-site wells
Huang et al. 2019	China<br>1 farm (joint with fish farm)<br>12,000 animals	Onsite facility wells used for drinking water for farm workers<br>3 wells on farm<br>Control site: None	DNA extracted from groundwater<br>qPCR for tetracycline (tetA, tetB, tetC, tetD, tetE, tetG, tetH,tetK, tetL, tetY, tetZ, tetA-P; tetM, tetO, tetQ, tetS, tetW, tetX) and sulfonamide (sul1, sul2) resistance genes	All tet genes and sul1 detected in all wells, sul2 not detected in wells<br>Most abundant ARG in wells: tetB<br>Abundance of ARGs one order of magnitude higher in wet vs. dry season ($p<0.01$)
Gao et al. 2020	China<br>Unspecified number of farms<br>2,000 sows and 48,000 piglets/year	Onsite and offsite private wells and facility wells<br>13 private wells in village with farms (distance from farms unspecified)<br>5 facility wells on swine farms<br>Control site: 4 wells in village with no swine farms	DNA extracted from groundwater<br>qPCR for sulfonamide (sul1, sul2), tetracycline (tetC, tetG, tetH, tetO, tetW, tetB/P), macrolide (ermA, ereA), chloramphenicol (cmlA, floR, fexA, cfr), quinolone (qnrD, qnrS), streptomycin (aadA), beta-lactam (blaTEM) resistance genes and integrons (intI1, intI2)	Absolute abundance of ARGs and integrons highest in facility wells on swine farms, similar in wells in villages with vs. without swine farms<br>Relative abundance of ARGs similar for all sites<br>Most common ARGs in wells: sul2 (99%), sul1 (40%), then tetC, tetG, floR, cmlA, aadA
**Poultry**				

**High-income countries**				
Hubbard et al. 2020	USA<br>9 farms<br>>1M chickens, >42,000 turkeys	Onsite and offsite private wells and facility wellls<br>10 private wells adjacent (0.5-1.6 km) to chicken farms<br>3 facility wells on turkey farms<br>Control site: 1 private well in watershed with no poultry farm but receiving wastewater discharge and likely swine manure	*Enterococci, Staphylococci, Lactobacilli* on plates with methicillin and sulfamethazine, then oxacillin<br>DNA extracted from oxacillin-resistant isolates for 16s rRNA sequencing<br>DNA extracted from groundwater<br>PCR for for beta-lactam (blaMOX, blaACC, blaFOX, blaDHA, blaEBC, blaIMP-1, blaNDM, blaKPC, blaVIM, blaOXA-48, blaTEM, blaCMY-2, blaCTX-M), tetracycline (tetM), colistin (mcr1) and macrolide-lincosamide-streptogramin B (ermB) resistance genes	Groundwater samples showed resistance to methicillin and oxacillin<br>77% (10/13) of samples had 1+ of 16 targeted ARGs<br>One groundwater sample had the mcr-1 gene<br>Contamination more frequent in watersheds with turkey farms than in poultry farms ($p<0.05$)<br>Contamination most frequent in control watershed with no poultry farms
Alsalah et al. 2003	Saudi Arabia<br>1 farm<br>Unspecified number of animals	Offsite wells of unspecified use type<br>3 wells <20 km from farm<br>Control site: 5 wells >20 km from farm	Bacterial growth on plates with meropenem and ceftazidime<br>*Pseudomonas aeruginosa* isolates tested for susceptibility to ampicillin, kanamycin, gentamicin, erythromycin, trimethoprim, sulfamethoxazole, tetracycline, ciprofloxacin, ceftazidime	No ARB detected in any well
Furtula et al. 2013	Canada<br>Unspecified number of farms and animals	Offsite wells of unspecified use type<br>28 wells in area of intensive poultry farming (distance from farms unspecified)<br>Control site: 1 well in residential area	*Enterococcus* isolates tested for susceptibility to chloramphenicol, ciprofloxacin, daptomycin, erythromycin, gentamicin, kanamycin, lincomycin, linezolid, nitrofurantoin, penicillin, streptomycin, Synercid® (quinupristin/ dalfopristin), tetracycline, tigecycline, tylosin, vancomycin	100% of isolates from wells at impacted sites resistant to 2+ antimicrobials<br>No *Enterococcus* detected in residential well at control site
**Low- and middle-income countries**				
Wang et al. 2017	China<br>1 farm<br>Unspecified number of animals	Onsite facility wells<br>1 well within layer<br>3 irrigation wells 8-25 m away from layer<br>Control site: None	Bacterial isolates from groundwater tested for susceptibility to norfloxacin hydrochloride, florfenicol, neomycin, amoxicillin, doxycycline hydrochloride, colistin	No bacteria isolated from wells 14-25 m away<br>Isolates resistant to neomycin, amoxicillin, doxycycline hydrochloride, colistin
**Cattle**				
**High-income countries**				
Li et al. 2014	USA<br>2 farms<br>Unspecified number of animals	Onsite monitoring wells<br>4 wells on 2 farms<br>Control site: None	*E. coli* isolates tested for susceptibility to amikacin, amoxicillin-clavulanic acid, ampicillin, cefoxitin, ceftiofur, ceftriaxone, chloramphenicol, ciprofloxacin, gentamicin, kanamycin, nalidixic acid, streptomycin, sulfisoxazole, tetracycline, trimethoprim/ sulfamethoxazole	25% (1/4) of isolates resistant to ceftriaxone and tetracycline and intermediate resistant to chloramphenicol<br>Higher resistance in February than in April and Sept-Oct
Li et al. 2015	USA<br>8 farms<br>Unspecified number of animals for sampled farms<br>1.7 million cows on 1,500 dairy	Onsite and offsite private wells and monitoring wells<br>46 monitoring wells immediately downstream of manure-treated fields, waste lagoons and corrals<br>5 domestic wells on farm<br>132 domestic or small community	*E. coli* and *Enterococcus* isolates tested for susceptibility with gram-negative Sensititre plate (CMV2AGNF) and gram-positive Sensititre plate (CMV3AGPF), respectively	64% of *E. coli* isolates and 86% of *Enterococcus* isolates resistant to 3+ antibiotics; prevalence of resistance not different between onsite monitoring wells vs. onsite domestic wells or between offsite wells <2.4 km vs. >2.4 km from dairy farm<br>*E. coli* predominantly resistant to azithromycin, chloramphenicol, trimethoprim/sulfamethoxazole, tetracycline<br>*Enterococcus* predominantly resistant to tigecycline, quinupristin/ dalfopristin, linezolid, chloramphenicol, erythromycin, ciprofloxacin,

	farms in study area	wells <2.4 km from dairy farm<br>Control site: 68 domestic or small community wells >2.4 km from a dairy farm<br>All offsite wells located near croplands likely receiving manure and near septic tanks		tetracycline
Guo et al. 2021	USA<br>1 farm<br>160 animals	Onsite wells of unspecified use type<br>1 well next to barn<br>1 well in farmland receiving manure (1 km from barn)<br>Control site: None for groundwater	DNA extracted from groundwater<br>qPCR for 113 ARGs, 21 mobile genetic elements	No ARGs in well next to barn<br>Multidrug resistance genes mexF and qacEΔ1 detected in well within farmland receiving manure<br>Relative abundance of ARGs in well higher than surface waters further from farm and fields receiving manure
**Mixed animals**				
**High-income countries**				
Economides et al. 2012	Cyprus<br>Unspecified number of farms and number of cattle, sheep, goat	Onsite wells of unspecified use type<br>Unspecified number of wells on farm<br>Control site: None	*Salmonella* and *E. coli* isolates tested for susceptibility to sulfamethoxazole, gentamicin, ciprofloxacin, ampicillin, cefotaxime, ceftazidime, tetracycline, streptomycin, trimethoprim, chloramphenicol, nalidixic-acid	48% (13/27) of *Salmonella* isolates resistant to 1+ antibiotic, 11% (3/27) resistant to 4+ antibiotics<br>30% (3/10) of *E. coli* isolates resistant to 1+ antibiotic, 20% (2/10) resistant to 3+ antibiotics<br>Mostly resistant to sulfamethoxazole, then tetracycline and streptomycin
Blauth et al. 2007	USA<br>11 swine and cattle farms<br>2,800-7,444 swine and 0-250 cattle per farm	Onsite facility wells<br>11 wells on 11 farms<br>Control site: None (for groundwater)	*E. coli* isolates tested for susceptibility to amikacin, ampicillin, amoxicillin/ clavulanic acid, ceftriaxone, chloramphenicol, ciprofloxacin, trimethoprim/ sulfamethoxazole, cefoxitin, gentamicin, nalidixic acid, sulfisoxazole, streptomycin, tetracycline, ceftiofur<br>*Enterococcus* isolates tested for susceptibility to chloramphenicol, erythromycin, penicillin, quinupristin/dalfopristin, tetracycline, vancomycin, ciprofloxacin, linezolid, nitrofurantoin, gentamicin, streptomycin, kanamycin, daptomycin, flavomycin, lincomycin, tigecycline, tylosin tartrate	100% (4/4) of *E. coli* isolates from wells resistant to tetracycline but no other antibiotics<br>80% (4/5) of *Enterococcus* isolates from wells resistant to 3 antibiotics, 20% (1/5) resistant to 4 antibiotics
**Low- and middle-income countries**				
Gu et al. 2022	China<br>101 swine farms, 52 poultry farms, 73 cattle farms<br>Unspecified number of animals	Onsite wells of unspecified use type<br>208 wells on farms<br>Control site: None	Plates with meropenem to isolate carbapenem-resistant *Enterobacteriaceae* (CRE)<br>CRE isolates tested for susceptibility to amikacin, cefotaxime, ceftazidime, ciprofloxacin, colistin, fosfomycin, nitrofurantoin, imipenem, meropenem, piperacillin/tazobactam, tigecycline, tetracycline<br>DNA extracted from CRE isolates<br>PCR for carbapenem (blaKPC, blaNDM, blaOXA-48, blaVIM, blaIMP) resistance genes<br>Whole genome sequencing	5% (11/208) of wells had CRE<br>- 16% (7/44) on poultry farms<br>- 3% (3/97) on swine farms<br>- 1.5% (1/67) on cattle farms<br>CRE more prevalent on poultry farms than on swine farms (p=0.011) and cattle farms (p=0.006), similar on swine and cattle farms (p=0.459)<br>100% of CRE isolates resistant to >2 antibiotics and resistant to cefotaxime, ceftazidime, tetracycline, ciprofloxacin, meropenem, imipenem<br>Most common ARGs in wells: blaNDM-5, then blaNDM-7, blaKPC-2, blaNDM-1, blaNDM-9
